# Volumetric Wear of Highly Cross-Linked Polyethylene Sockets in Total Hip Arthroplasty Differs Among Brands: A Retrieval Study

**DOI:** 10.3390/jfb17100522

**Published:** 2026-10-16

**Authors:** Peter Wahl, Valerio Porrati, Michel Schläppi, Hannes Kuttner, Florian Sax, Martin H. Resl, Sebastian Simon, Karina Hackl, Martina Winkelmann, Gabriela von Lewinski, E. Bailey Terhune, Roman Heuberger, Deborah J. Hall, Robin Pourzal

**Affiliations:** 1 Department Biomedical Engineering, University of Basel, Hegenheimermattstrasse 167 B/C, 4123 Allschwil, Switzerland; 2 ARTORG Centre for Biomedical Engineering Research, Faculty of Medicine, University of Bern, 3008 Bern, Switzerland; 3 Endo-Team, Birshof Hospital, 4142 Muenchenstein, Switzerland; 4 Department of Orthopedic Surgery, Rush University Medical Center, Chicago, IL 60612, USA; valerio.porrati@unibas.ch (V.P.); bailey.terhune@rushortho.com (E.B.T.); deborah_hall@rush.edu (D.J.H.); robin_pourzal@rush.edu (R.P.); 5 Biomedical Engineering, Polytechnic University of Milan, 20133 Milan, Italy; 6 Division of Orthopaedics and Traumatology, Cantonal Hospital Winterthur, 8400 Winterthur, Switzerland; michel.schlaeppi@ksw.ch; 7 Department of Orthopaedic Surgery and Traumatology, Cantonal Hospital Chur, 7000 Chur, Switzerland; hannes.kuttner@ksgr.ch; 8 Division of Endoprosthetics, Orthopaedic Clinic Markgroeningen, 71706 Markgroeningen, Germany; florian.sax@rkh-gesundheit.de; 9 Centre for Musculoskeletal Surgery, Charité University Medicine Berlin, 10117 Berlin, Germany; martin.resl@charite.de; 10 Michael Ogon Laboratory for Orthopaedic Research, Orthopaedic Hospital Vienna-Speising, 1130 Vienna, Austria; sebastian.simon@oss.at (S.S.); karina.hackl@oss.at (K.H.); 11 Center for Anatomy and Cell Biology, Medical University of Vienna, 1090 Vienna, Austria; 12 Department of Orthopaedic Surgery and Traumatology, Cantonal Hospital Frauenfeld, 8500 Frauenfeld, Switzerland; martina.winkelmann@stgag.ch; 13 Department of Traumatology, Hannover Medical School, 30625 Hannover, Germany; vonlewinski.gabriela@mh-hannover.de; 14 RMS Foundation, 2544 Bettlach, Switzerland; roman.heuberger@rms-foundation.ch

**Keywords:** total hip arthroplasty, THA, polyethylene, highly cross-linked polyethylene, HXLPE, wear

## Abstract

Outcomes after total hip arthroplasty (THA) remain limited by wear and wear particle reaction. Although highly cross-linked polyethylene (HXLPE) mitigated this issue, there are major differences among the various brands available. This study quantified, for the first time, the volumetric wear on the retrievals from various HXLPE brands, using an optical coordinate measuring machine. Patient- and implant-related factors and component orientation were considered. As of 30.06.2025, 104 HXLPE sockets were available, and 79 could be analysed, excluding those too severely deformed/damaged. The median (range) time in situ was 8.7 (2.1–22.1) years. Wear volume and volumetric wear rate were 91 (1–674) mm^3^ and 9 (0–130) mm^3^/year, respectively. Highcross (*n* = 56, Medacta) experienced approximately five times more wear (*p* < 0.001) than Durasul (*n* = 17, Zimmer Biomet). The other brands (*n* = 6) were too small in group size at this stage for statistical analysis. The correlation (R^2^ = 0.94) between linear and volumetric wear was excellent, but high wearing retrievals had a strong impact on this analysis, whereas low wear retrievals showed high variability. Differences in wear were mostly driven by the brand, while other parameters had only a minor impact. This is the first retrieval study comparing volumetric wear among various brands of retrieved HXLPE bearings. Not all HXLPE bearings should be considered equal. As the cohort grows, further parameters and brands may be integrated.

## 1. Introduction

Wear remains one of the main issues affecting long-term outcomes after total hip arthroplasty (THA) due to inflammatory reactions with wear particles within the periprosthetic tissues and subsequent development of osteolysis and implant loosening [1,2,3,4,5,6]. Technical solutions have been developed in two directions to mitigate this issue: either hard-on-hard bearings (metal-on-metal or ceramic-on-ceramic) or using highly cross-linked polyethylene (HXLPE) sockets. The latter has emerged as the gold standard over the last 15 years [7,8,9] due to its excellent wear performance and because hard-on-soft bearings are more forgiving and have shown excellent long-term results, while lacking other risks such as fracture or squeaking, as reported for some ceramic bearings, or adverse tissue responses to cobalt-containing wear debris, as seen with some metal-on-metal bearings.

The introduction of HXLPE significantly reduced long-term revision rates after THA [10,11,12], but wide differences in revision rates between individual subcategories were identified, which are, at least in part, related to manufacturer-specific processing parameters (e.g., type and dose of irradiation) [13]. While HXLPE greatly reduced volumetric wear in THA compared to conventional, ultrahigh-molecular-weight polyethylene (CPE) [14,15,16,17,18], the wear particles produced are smaller and may induce a stronger immune cell response at equal volume [19,20,21,22,23,24]. Even minor differences in manufacturing steps and processing of HXLPE may have relevant consequences regarding long-term mechanical and tribological behaviour [13,25,26,27]. In addition, isolated cases of early failure of well-positioned THA with HXLPE bearings have been reported, with no indication that patient factors had contributed to the failure [28,29,30,31,32,33]. Thus, the question arises as to whether implant-specific factors, such as wear rates, affect the overall outcome. A recent study of the in vitro wear behaviour of various HXLPE types reported a wide range of wear rates among brands [27]. Thus, contrary to the expectation of most orthopaedic surgeons, not all HXLPE bearings appear to be equal. So far, only data from simulator studies [17,18] or radiologic estimates of in vivo wear [15,34,35,36,37,38] have been available. Volumetric wear assessments of retrieved HXLPE sockets have been limited to isolated case studies [34,39,40,41].

This study aimed to quantify the volumetric wear of surgically retrieved HXLPE THA sockets from various manufacturers, providing benchmark values, while integrating known and suspected clinical confounding factors into the analysis. To the best of our knowledge, this represents the first description of volumetric wear in a series of retrieved HXLPE sockets from various brands. Our hypothesis was that HXLPE sockets would exhibit broad variation in wear rate, in part explained by manufacturer-specific processing parameters and patient-specific risk factors.

## 2. Methods

### 2.1. Patients and Bearing Retrieval 

Acetabular HXLPE bearings (liners and cups, with the corresponding ball head) from a continuous series of THA revisions performed since August 2022 at participating tertiary reference centres in Switzerland, Germany, and Austria were included if retrieved after more than two years in vivo. Dual mobility bearings and hooded liners were excluded. Furthermore, bearings were excluded from this study if severe iatrogenic damage, lack of unworn areas, or severe plastic deformation hindered the digital reconstruction of the initial surface and, thus, accurate wear quantification.

Clinical data such as patient age, height, weight, American Society of Anesthesiology (ASA) class, time in situ, component type and fixation, component orientation (as measured on conventional radiographs [42] respectively CT scans if available [43,44]), and reason for revision was obtained from electronic patient files and a review of available radiologic imagery.

### 2.2. Wear Quantification

**Figure 1:**
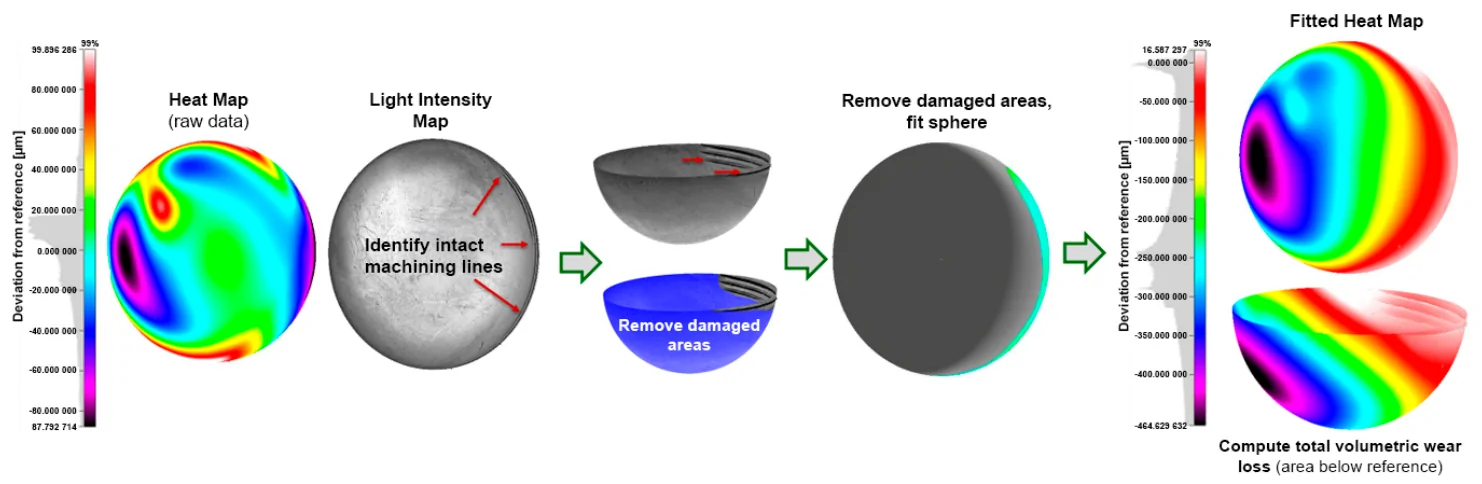
Illustration of steps for determination of volumetric wear on sockets. Measurements were capture using 5-axis optical coordinate measuring machine OrthoLux (RedLux, Romsey, United Kingdom). Digital reconstructions and wear analysis were performed with RedLux Studio Software (version 7.1).

Volumetric wear of the polyethylene sockets as well as the exact diameter of the socket and of the femoral head were assessed quantitatively with a 5-axis optical coordinate measuring machine with a white-light chromatic confocal sensor (OrthoLux, RedLux, Romsey, UK). All sockets were scanned within 3 to 6 months after retrieval. The bearing surface was scanned with 1080 data points per rotation and three rotations per degree. The resolution along the *z*-axis was 20 nm [45]. Digital reconstruction of the original sphere and wear analysis were performed with RedLux Studio software (version 7.1) using a previously validated methodology [46]. Unworn areas exhibiting initial machining marks were identified using light intensity maps, and worn areas on the bearing surface were removed. A sphere was fitted to the remaining unworn regions using a linear least-squares fit. Worn regions were reinserted in reference to the new set origin of the original sphere. Wear was defined as areas below the height range of the machining line topography within the unworn areas, and wear volume was computed accordingly (Figure 1). Localised damage caused by screws used for removal of the inlay was removed from wear measurements, considering the defect spherical. The femoral head was measured in the same manner. While no to minimal wear occurred on the metal or ceramic femoral heads, areas with scratches or deposits were removed before fitting a sphere and determining the exact diameter. The radial clearance of the bearing was computed as the radial difference between the two surfaces. Fitting, volumetric material loss assessment and determination of the diameter were performed in triplicate. Maximum linear wear was also extracted from wear maps for each socket using the 99th percentile value to avoid the influence of outliers (resulting, for example, from pits). All calculations were performed by the same person (V.P.).

### 2.3. Statistical Analysis

Scalar and normally distributed data are described using mean and standard deviation (SD), with comparison performed using Student’s *t*-test. Non-normally distributed scalar data are described using median and range, with comparison performed using the Mann–Whitney U-test. Categorical data are described in counts and percentages, with comparison performed using Fisher’s exact test. Multiple testing was addressed using the false discovery rate (FDR) correction. Multiple linear regression was used to identify determinants of wear. Statistical significance was accepted at a two-sided *p*-value < 0.05 for all analyses. The null hypothesis was that there was no difference between the various brands of HXLPE regarding volumetric wear. All statistical analyses were conducted using R version 4.4.2 (R Foundation for Statistical Computing, Austria), with the ggplot2 package for graphical illustration.

## 3. Results

As of 30.06.2025, a total of 103 retrievals were available (Table 1). All retrievals were collected in Switzerland and provided by three different centres, except three retrievals from Germany. In total, 24 had to be excluded (15/71 (21%) from Highcross, 4/21 (19%) from Durasul and 5/11 (45%) of the other brands): nine due to low wear in the range of initial shape deviations from the manufacturing process, six due to excessive deformation at retrieval, five due to severe iatrogenic damage, three due to excessively damaged in vivo prohibiting reliable measurements, and one liner due to not exhibiting sufficient undamaged surface area to enable digital reconstruction, leaving 79 components for analysis. The study cohort consisted of liners from the following manufacturers: 56 Highcross (Medacta, Castel San Pietro, Switzerland), 17 Durasul (Zimmer Biomet, Zug, Switzerland), two Altrx (DePuy, Zuchwil, Switzerland), two Marathon (DePuy), one XLPE (Smith & Nephew, Zug, Switzerland), and one XLPE (Meril, Bonn, Germany). Lot numbers were registered whenever identifiable. No two components shared the same lot number. For statistical comparison, groups with less than three liners were combined and labelled as “other brands”.

**Figure 2:**
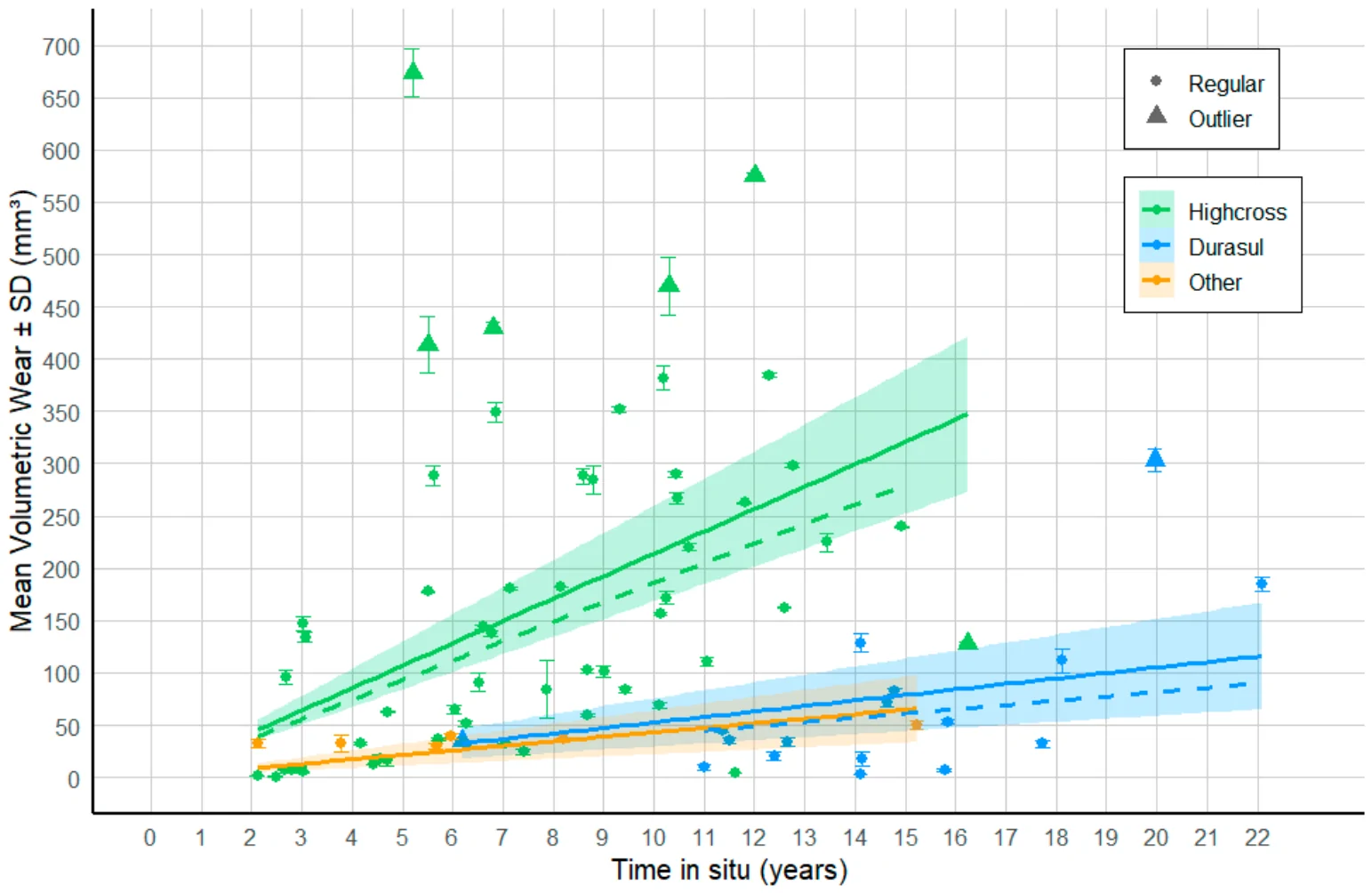
Mean volumetric wear (± standard deviation) over time in situ for Highcross, Durasul, and sockets from other brands (2 Altrx (DePuy), 2 Marathon (DePuy) 1 XLPE (Smith & Nephew), 1 XLPE (Meril)). Each data point represents mean wear of one implant, with error bars indicating standard deviation. Solid lines represent linear regression fits through the origin for each group using all data points. The shaded bands indicate the 95% CI of the respective regression slope. Of note, this does not correspond to the confidence interval of the data distribution, which was much larger, as the model explained only part of the variability (R^2^ = 0.615 for Highcross, R^2^ = 0.592 for Durasul and R^2^ = 0.847 for the other brands). Dashed lines show the regression fits after exclusion of the identified outliers (standardised residuals > |2| or Cook’s distance > 4/*n*). Triangles indicate outliers. Circular dots indicate regular data points.

**Figure 3:**
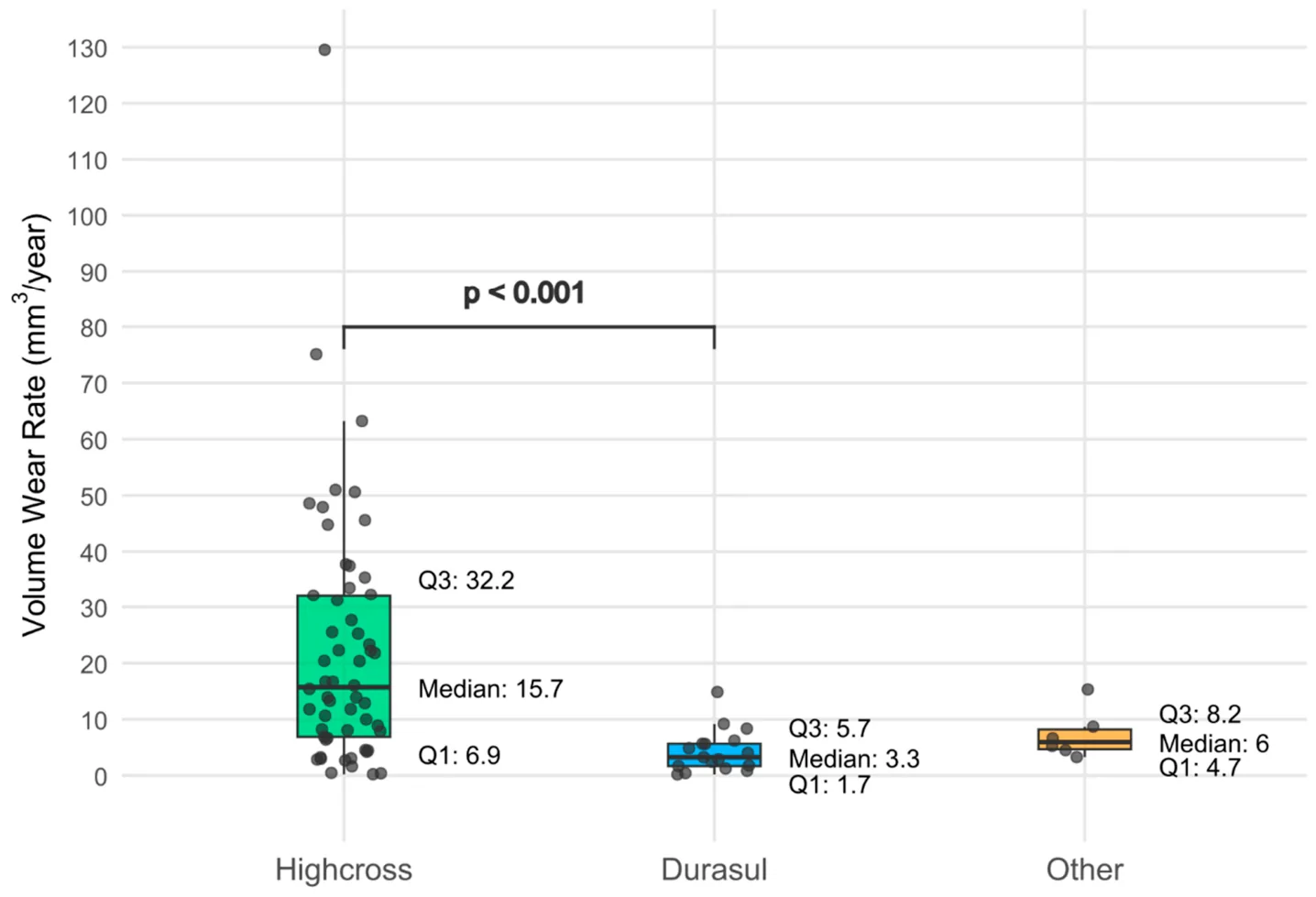
Boxplots comparing volumetric wear rates between sockets from various brands available. “Other” includes 2 Altrx (DePuy), 2 Marathon (DePuy) 1 XLPE (Smith & Nephew), and 1 XLPE (Meril). The horizontal line within the box represents the median, whereas the box delimitates the interquartile range (Q1–Q3). The whiskers represent 1.5× the interquartile range. Individual data points are added, jittered. The statistical differences between two groups were assessed using the Wilcoxon rank-sum test.

The median (range) time in situ was 8.7 (2.1 to 22.1) years. The general cohort exhibited a median (range) wear volume of 91 (1 to 674) mm^3^, corresponding to a median volumetric wear rate of 9 (0 to 130) mm^3^/year (Table 2). By group, the median (range) volumetric wear and wear rates were 138 (1 to 674) mm^3^ and 16 (0 to130) mm^3^/year for Highcross, 35 (3 to 297) mm^3^ and 3 (0 to 15) mm^3^/year for Durasul, and 35 (30 to 50) mm^3^ and 6 (3 to 15) mm^3^/year for the other brands. The volumetric wear over time is illustrated in Figure 2. In all groups, wear exhibited a positive correlation with time in situ. The wear rate of Highcross was approximately five times higher than that of Durasul (*p* < 0.001) (Figure 2 and Figure 3). The wear rate did not differ significantly between isolated polyethylene sockets and liners retrieved together with the metalback cup (*p* = 0.41).

**Figure 4:**
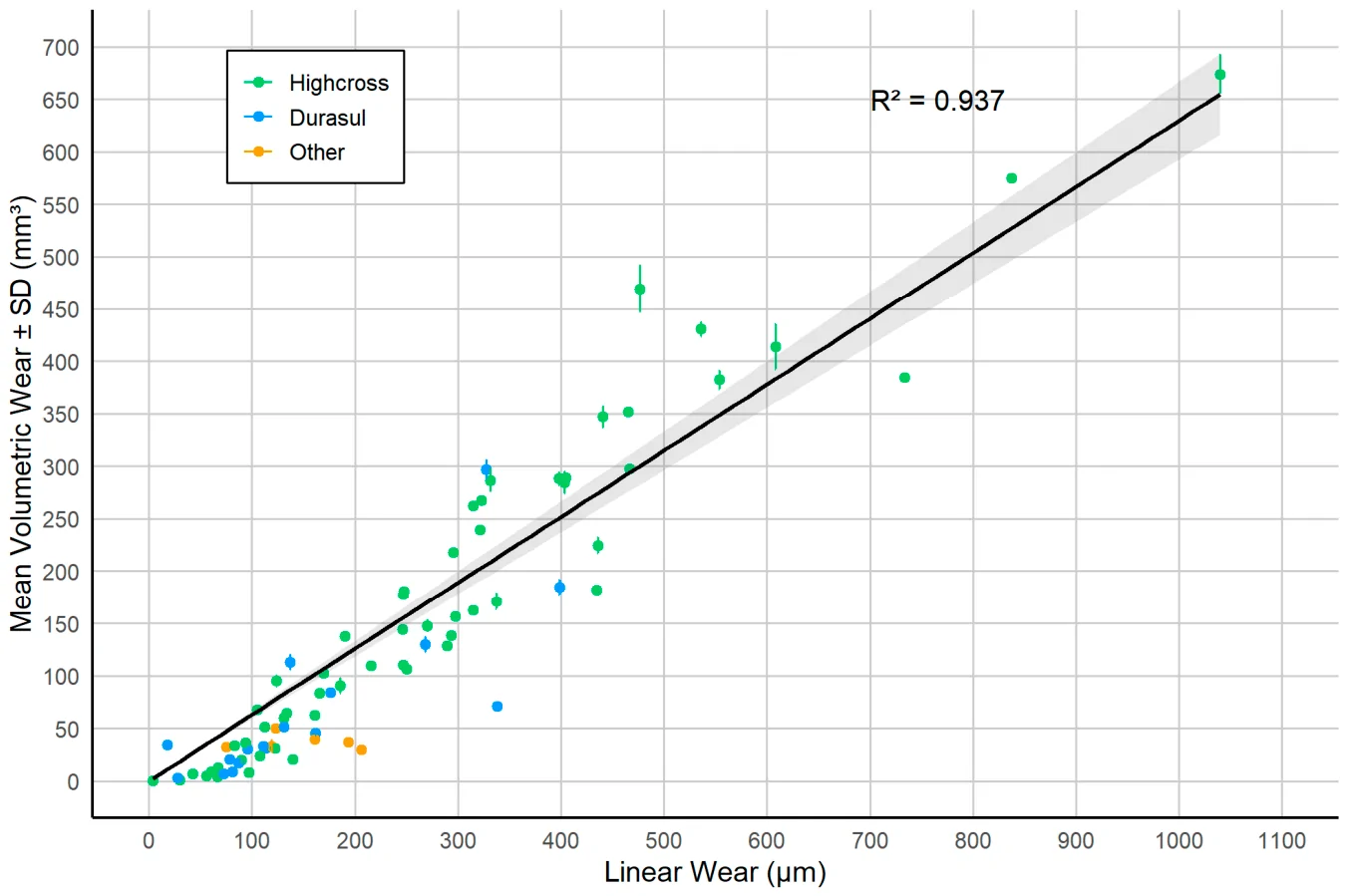
Relation between the linear wear and the volumetric wear, as measured from the retrievals. The shaded area marks the 95% confidence interval of the linear regression (black line). Note the high variability particularly for low to medium wear, despite an overall R^2^ = 0.937, which was largely influenced by the retrievals with very high wear. The difference among groups was close to statistical significance but did not reach the set threshold (*p* = 0.052).

The median linear wear and wear rate were 161 µm and 19 µm/year for the entire cohort. The median linear wear rate for Highcross was close to 3× higher than for Durasul at 27.4 µm/year and 11.7 µm/year, respectively. There was a trend towards differences among the various subgroups (*p* = 0.052). The correlation of linear wear to volumetric wear is illustrated in Figure 4. While there was an excellent correlation overall (R^2^ = 0.94), this must be considered with caution, because retrievals with high wear had a large impact on this statistical analysis, whereas the retrievals with low wear showed very high variability.

Further analysis of the potential determinants of wear was performed only for the two most represented HXLPE brands, Highcross and Durasul, considering the small number of retrievals from the other brands available so far. The initial multiple linear regression resulted in time in situ (*p* = 0.014), patient age (*p* = 0.038), and HXLPE manufacturer (*p* < 0.001) as significant determinants of volumetric wear. Given the brand’s significant influence (Figure 3), multiple linear regression was performed separately for both Highcross and Durasul. Time in situ was a significant determinant of volumetric wear for both groups, with a more significant effect for Highcross (*p* = 0.005) than for Durasul (*p* = 0.023) (Table 3). Weight was highly significantly associated with volumetric wear as well as with the wear rate of Durasul (*p* = 0.002) (Table 4). A higher ASA score (III & IV) was negatively associated with the volumetric wear (*p* = 0.041) and wear rate (*p* = 0.023) of Highcross.

## 4. Discussion

This study aimed to establish benchmark in vivo wear rate values for THA HXLPE bearings from a cohort of surgically retrieved sockets from various brands and to identify determinants of wear, including HXLPE brand. We found a broad range of wear, mostly driven by the HXLPE brand, thereby confirming our initial hypothesis. However, this conclusion is based on only the two HXLPE brands that were available in sufficient numbers at this time of this ongoing study. Wear rate was also, to a lesser degree, driven by time in situ, ASA class, and patient weight.

The introduction of HXLPE sockets in THA was a major technological advancement, resulting in reduced wear and much-improved implant longevity [15,34,35,36,38]. This success has been in large part due to improved wear performance. Our retrieval study exhibited an overall wear rate of 9 mm^3^/year, compared to 64 mm^3^/year in contemporary CPE liners sterilised in an inert environment [47], to 100 mm^3^/year in historic CPE liners sterilised in air [47], and to 41 mm^3^/year in well-functioning liners made from historic CPE and retrieved post mortem [48,49]. A previous study reported a wear rate of 10 mm^3^/year for early-generation HXLPE liners (Longevity, Zimmer Biomet, *n* = 11), which is close to our reported value. However, for Durasul—a product comparable to Longevity—the wear rate is more than three times lower, at only 3 mm^3^/year. Other volumetric HXLPE retrieval wear data are limited to case studies [10,39,40,41], with reported values that fall within the range presented here. However, Highcross showed a large range of volumetric wear rates (median 16, range 0–130 mm^3^/year). While some of these bearings performed similarly to other brands, many experienced higher wear, matching and, in some cases, even exceeding the wear rates for CPE [47,48,49]. Given the lack of large-scale retrieval studies on HXLPE wear, the better wear performance of HXLPE compared to CPE has been primarily established through simulator tests [18,24,50,51,52]. However, simulator studies are limited, representing a single, standardised, ideal scenario differing from in vivo conditions. In particular, patient-specific motion, loading, and activity level dictate wear rate [53], while component impingement and subluxation also affect a large proportion of patients receiving THA, with implications for the wear behaviour [54,55]. Furthermore, the in vivo alteration of the bearing surface due to oxidation or deposition and absorption of organic species from the pseudo-synovial fluid affects tribological behaviour [56,57]. Affatato et al. reported wear rates from hip wear simulator studies of HXLPE (75 kGy) sockets of 6.4 mg/million cycles [58]. Assuming an average number of daily steps of 5000 to 7500 and an HXLPE density of 0.934 g/mm^3^, which would equal 11.7 to 17.5 mm^3^/year. Therefore, this predicted value lies within our reported range, although the sockets in this study were irradiated with nominal doses of 100 and 95 kGy for Highcross and Durasul, respectively [27].

In vivo wear assessment is mostly performed radiographically [15,34,35,36,38]. This method only provides a linear wear value, requiring extrapolations for volumetric wear assessment, further decreasing precision [59]. The linear wear rate of the entire cohort was 19 µm/year in our study, compared to ~30 (range) µm/year in other studies [60]. However, when separating wear rates between the two main brands represented here, only the rates of Highcross match previous radiographic data, while Durasul liners exhibited an almost threefold lower linear wear rate. It must be considered that the radiographic assessment of wear inherently lacks precision—particularly in lower-wearing HXLPE liners—due to constraints involving resolution, inconsistent positioning during imaging, and overestimation of wear caused by the initial bedding-in process (driven in part by creep). Measurements with the optical CMM have excellent spatial resolution (20 nm in the *z*-axis [45]) and are considerably more precise than any radiologic evaluation. In our study, all wear measurements were performed by the same person, avoiding inter-observer variability.

These preliminary results identify important differences in the volumetric wear and wear rates between the two brands with a sufficient group size allowing a statistically meaningful comparison (Figure 2 and Figure 3, Table 2 and Table 3), Highcross retrievals showing wear rates about five times higher as Durasul, with considerably greater variability for Highcross. These results corroborate a previous in vitro wear study that showed a similar difference in wear rate under equal testing conditions [27]. Considering the number of parameters known or suspected to influence wear in THA, it is surprising that the brand-specific differences remain so important. As no two retrievals shared the same lot number, our results can be considered representative of the whole product, not being linked to regional phenomena. Manufacturing details available for the HXLPE included in this study and their potential influence on wear resistance are discussed more in detail in a previous publication [27]. It must however be pointed out that most manufacturing details are usually not disclosed by the manufacturers. The same study also suggested that the wear rates of other HXLPE brands are either equal to those of Durasul or fall between the rates of Durasul and of Highcross. However, a larger study cohort is needed to confirm these findings and to integrate additional clinical risk factors. Differences in HXLPE are driven by multiple processing factors during manufacturing (e.g., the type and dose of irradiation, single-dose vs. sequential irradiation, annealing vs. remelting as thermal treatment post-irradiation) [25,26,27,61]. Therefore, not all HXLPE are same, which also affects wear behaviour and potentially the periprosthetic tissue response. While the HXLPE brand was the dominant determinant of wear, other factors, such as time in situ, ASA score, and weight, were also significant, all of which are surrogate measures of patient-specific activity and loading.

A relevant proportion of these cases were revised for aseptic loosening (Table 1), which may have been associated with a wear particle reaction [1,41]. The occurrence of wear-particle-induced inflammatory reactions, which can lead to osteolysis and component loosening [2,3], does not depend on volumetric wear only. Particle size and shape also play a relevant role, with smaller particles potentially inducing a stronger macrophage reaction at equal volume [19,20,21,22,23,24]. HXLPE typically produces smaller particles than CPE [21,24,28,41]. Thus, even if an acceptable wear rate threshold was established for CPE [1], it would likely not be valid for modern bearings. Ongoing studies will characterise the wear particles according to HXLPE brand and will also quantify the macrophage reaction within periprosthetic tissues. As the cohort grows, it will also be possible to meaningfully integrate the reasons for revision into the analysis.

This study has several limitations. The collection of retrievals was limited by regional implant preferences and the exclusion of approximately one-fifth of the cases due to damage, making the digital reconstruction of the original surface impossible. Therefore, this ongoing study could only conduct a statistically meaningful comparison between Highcross and Durasul. However, a much larger proportion of Highcross was revised at the medium term than for Durasul (Figure 2 and Table 1). A larger and more diverse study cohort is needed to confirm the results and to determine potential other differences in wear behaviour between designs and polyethylene types. It also has to be acknowledged that some liners exhibited removal damage that did not lead to exclusion from this study. Liners are commonly removed with a screw that is placed in the rim. While the screw does not directly damage the surface, it causes material to be elevated within the bearing surface. Implants with such artifacts, which occurred independent of brand, would result a slight underestimation of wear volume and were only included if the screw bulge was not located within the main wear area. The analysis of the clinical determinants of wear was also limited by cohort size (Figure 3). A larger cohort would provide greater statistical power to detect the potential impact of other patient-related, surgical, and implant design factors, which may have been obscured by the brand’s dominant influence in the current cohort. Furthermore, wear is primarily a function of use [53], but it is difficult or impossible to retrospectively assess patient activity in revision arthroplasty with follow-up examinations spaced by years. In the meantime, sex, age, and ASA score can serve as proxy measures to some degree. Finally, this study only determined in vivo wear rates but did not allow direct correlation of wear with implant failure. However, the availability of volumetric wear data lays the foundation for future research on wear thresholds and the correlation between particle size and periprosthetic inflammation, osteolysis, and ultimately implant failure. That these differences may impact revision rates has not been yet proven, but differences in medium-term cumulative revision rates can already be observed in the Swiss Arthroplasty Registry SIRIS, which anecdotally reports revision rates by bearing for the main component brands [62].

## 5. Conclusions

This study provides preliminary benchmark values of the volumetric wear of contemporary HXLPE sockets in THA. At an early stage, this ongoing study identified major differences in wear resistance among major HXLPE brands. Brand-specific differences in HXLPE resulting from various manufacturing processes may be more important than commonly accepted, and their clinical impact with respect to mid- to long-term implant failure needs to be investigated in more detail. All HXLPE brands should no longer be grouped in studies and registries, but a more detailed analysis is necessary, considering manufacturing specificities or individual brands. As the study cohort grows, more confounding factors may be considered, and our understanding of their influence on the volumetric wear of HXLPE sockets in THA can be refined.

## Figures and Tables

**Table 1:** A summary of the specifications of the retrievals available as well as of the associated clinical data.

Parameter	Subcategory	All (*n* = 79)	Highcross (*n* = 56)	Durasul (*n* = 17)	Statistics	Other Brands (*n* = 6) *
Male:female *n*:*n* (%:%)		44:35 (56%:44%)	33:23 (59%:41%)	7:10 (41%:59%)	0.198	4:2 (67%:33%)
Right:left *n*:*n* (%:%)		42:37 (53%:47%)	30:26 (54%:46%)	10:7 (59%:41%)	0.703	2:4 (33%:67%)
Age at implantation, years, median (range)		63 (27–92)	63 (27–92)	64 (36–76)	0.553	65 (44–79)
Time in situ, years, median (range)		8.7 (2.1–22.1)	7.3 (2.1–16.2)	14.2 (6.2–22.1)	<0.001	5.8 (2.1–15.2)
Age at revision, years, median (range)		74 (38–97)	69 (38–97)	77 (56–92)	0.035	75 (50–87)
ASA class at revision, *n* (%)	- I & II - III & IV	40 (51%) 39 (49%)	33 (59%) 23 (41%)	3 (18%) 14 (82%)	0.005	4 (67%) 2 (33%)
Weight at revision, kg, median (range)		73 (30–145)	74 (52–119)	71 (30–145)	0.561	74 (65–99)
BMI at revision kg/m^2^ median (range)		26.0 (13.3–42.4)	25.7 (17.4–38.6)	26.7 (13.3–42.4)	0.865	26.2 (21.5–29.2)
Radiologic cup inclination, degrees median (range)		42 (30–60)	42 (30–51)	43 (31–60)	0.136	41 (27–55)
Radiologic cup anteversion, degrees median (range)		15 (0–38)	14 (7–31)	21 (0–38)	0.002	21 (18–37)
Cup inside Lewinnek’s safe zone, *n*:*n* (%:%)		58:21 (73%:27%)	45:11 (80%:20%)	7:10 (41%:59%)	0.071	3:3 (50%:50%)
Socket separate:with cup, *n*:*n* (%:%)		37:42 (47%:53%)	24:32 (43%:57%)	11:6 (65%:35%)	0.114	2:4 (33%:67%)
Bearing diameter, *n* (%)	- 28 mm - 32 mm - 36 mm - 40 mm	14 (18%) 54 (68%) 10 (13%) 1 (1.3%)	11 (20%) 41 (73%) 4 (7.1%) 0 (0%)	3 (18%) 12 (71%) 2 (12%) 0 (0%)	0.805	0 (0%) 1 (17%) 4 (67%) 1 (17%)
Ball head material, *n* (%)	- CoCr - alumina - ZTA ** - oxidised zirconium	11 (14%) 8 (10%) 59 (75%) 1 (1.3%)	4 (7.1%) 1 (1.8%) 51 (91%) 0 (0%)	6 (35%) 7 (41%) 4 (24%) 0 (0%)	<0.001	1 (17%) 0 (0%) 4 (67%) 1 (17%)
Neck length, *n* (%)	- S/ + 1.5 - M/ + 5 - L/ + 8.5 - XL/ + 12	9 (11%) 52 (66%) 14 (18%) 4 (5%)	7 (13%) 38 (68%) 10 (18%) 1 (2%)	1 (5%) 11 (65%) 3 (18%) 2 (12%)	0.359	1 (17%) 3 (50%) 1 (17%) 1 (17%)
Neck angle ***, *n* (%)	- standard - lateralised	56 (71%) 23 (29%)	38 (68%) 18 (32%)	13 (76%) 4 (24%)	0.498	5 (83%) 1 (17%)
Cup cemented, *n* (%)		1 (1.3%)	0 (0%)	1 (5.9%)	0.233	0 (0%)
Stem cemented, *n* (%)		13 (16%)	9 (16%)	2 (12%)	0.664	2 (33%)
Any component cemented *n* (%)		13 (16%)	9 (16%)	2 (12%)	0.664	2 (33%)
Stem antetorsion ****, ° median (range)		16 (−16 to 34)	16 (−4 to 34)	25 (−16 to 31)	0.508	15 (10–34)
reason for revision, *n* (%)	- infection - fracture acetabulum - osteolysis acetabulum - loosening cup - malposition cup - instability - wear - wear particle disease - malposition stem - fracture femur - osteolysis femur - loosening femur - delamination - heterotopic ossifications	7 (8.9%) 3 (3.8%) 10 (13%) 1 (1.3%) 17 (22%) 4 (5.1%) 3 (3.8%) 5 (6.3%) 1 (1.3%) 25 (32%) 18 (23%) 36 (46%) 2 (2.5%) 1 (1.3%)	5 (8.9%) 2 (3.6%) 9 (16%) 1 (1.8%) 8 (14%) 2 (3.6%) 1 (1.8%) 4 (7.1%) 0 (0%) 15 (27%) 14 (25%) 27 (48%) 1 (1.8%) 0 (0%)	1 (5.9%) 1 (5.9%) 1 (5.9%) 0 (0%) 8 (47%) 0 (0%) 2 (12%) 1 (5.9%) 0 (0%) 9 (53%) 4 (24%) 6 (35%) 0 (0%) 1 (5.9%)	0.151	1 (17%) 0 (0%) 0 (0%) 0 (0%) 1 (17%) 2 (33%) 0 (0%) 0 (0%) 1 (17%) 1 (17%) 0 (0%) 3 (50%) 1 (17%) 0 (0%)

* 2 Altrx (DePuy), 2 Marathon (DePuy) 1 XLPE (Smith & Nephew), 1 XLPE (Meril). ** ZTA = zirconia-toughened alumina. *** “standard” includes stems with CCD of 135°, also corresponding to Fitmore stem type B, whereas “lateralised” includes stems with lateralised centre of rotation even if CCD remains at 135° and stem models with reduced CCD (125–128°). **** Available for 40 cases only.

**Table 2:** Results of component measurements and calculations of volumetric wear.

Parameter	All (*n* = 79)	Highcross (*n* = 56)	Durasul (*n* = 17)	Statistics	Other Brands (*n* = 6) *
radial clearance, mm, median (range)	0.14 (0.02–0.61)	0.13 (0.02–0.32)	0.18 (0.10–0.33)	<0.001	0.43 (0.04–0.61)
volumetric wear of socket, mm^3^, median (range)	91 (1–674)	138 (1–674)	35 (3–297)	0.012	35 (30–50)
volumetric wear rate of socket, mm^3^/year, median (range)	9 (0–130)	16 (0–130)	3 (0–15)	<0.001	6 (3–15)
linear wear of socket, µm, median (range)	186 (4–1’040)	247 (4–1’040)	113 (18–398)	0.019	142 (75–206)
linear wear rate of socket, µm/year, median (range)	24 (2–200)	30 (2–200)	8 (2–23)	<0.001	29 (8–36)

* 2 Altrx (DePuy), 2 Marathon (DePuy) 1 XLPE (Smith&Nephew), 1 XLPE (Meril).

**Table 3:** Results of multiple linear regression analysis of determinants of volumetric wear, separated for two main brands in the cohort, as there were large differences in volumetric wear between these brands.

Parameter	Coefficient	Standard Error	t-Stat	*p*-Value	Lower 95%	Upper 95%
**Highcross**						
Intercept	−54.408	115.773	−0.47	0.640	−287.00	178.00
Weight (per kg)	1.652	1.152	1.43	0.157	−0.66	3.96
Time in situ (per year)	16.197	5.552	2.92	0.005	5.05	27.30
ASA class III & IV vs. I & II (ref)	−83.055	39.586	−2.10	0.041	−163.00	−3.58
Lewinnek’s safe zone outside vs. inside (ref)	9.016	46.607	0.19	0.847	−84.60	103.00
**Durasul**						
Intercept	−191.234	48.399	−3.95	0.002	−297.00	−85.80
Weight (per kg)	1.923	0.492	3.91	0.002	0.85	2.99
Time in situ (per year)	8.862	3.396	2.61	0.023	1.46	16.30
ASA class III & IV vs. I & II (ref)	−26.632	32.044	−0.83	0.422	−96.40	43.20
Lewinnek’s safe zone outside vs. inside (ref)	16.111	22.999	0.70	0.497	−34.00	66.20

**Table 4:** Results of multiple linear regression analysis of determinants of volumetric wear rates, separate for two main brands in the cohort, as there were large differences in wear rates between these brands.

Parameter	Coefficient	Standard Error	t-Stat	*p*-Value	Lower 95%	Upper 95%
**Highcross**						
Intercept	9.665	15.315	0.63	0.531	−21.05	40.38
Weight (per kg)	0.181	0.178	1.02	0.313	−0.17	0.54
ASA class III & IV vs. I & II (ref)	−14.051	6.050	−2.32	0.024	−26.26	−1.84
Lewinnek’s safe zone outside vs. inside (ref)	5.540	7.319	0.76	0.453	−9.21	20.29
**Durasul**						
Intercept	−3.889	2.356	−1.65	0.123	−8.26	0.48
Weight (per kg)	0.113	0.030	3.77	0.002	0.05	0.18
ASA class III & IV vs. I & II (ref)	−1.127	1.952	−0.58	0.574	−5.46	3.21
Lewinnek’s safe zone outside vs. inside (ref)	1.261	1.428	0.88	0.393	−1.75	4.27

## Data Availability

The raw data supporting the conclusions of this article will be made available by the authors on request.
